# Heliorhodopsin Evolution Is Driven by Photosensory Promiscuity in Monoderms

**DOI:** 10.1128/mSphere.00661-21

**Published:** 2021-11-24

**Authors:** Paul-Adrian Bulzu, Vinicius Silva Kavagutti, Maria-Cecilia Chiriac, Charlotte D. Vavourakis, Keiichi Inoue, Hideki Kandori, Adrian-Stefan Andrei, Rohit Ghai

**Affiliations:** a Department of Aquatic Microbial Ecology, Institute of Hydrobiology, Biology Centre of the Academy of Sciences of the Czech Republic, České Budějovice, Czech Republic; b Department of Ecosystem Biology, Faculty of Science, University of South Bohemia, Branišovská, České Budějovice, Czech Republic; c Research Institute for Biomedical Aging Research, University of Innsbruck, Innsbruck, Austria; d European Translational Oncology Prevention and Screening (EUTOPS) Institute, Tirol, Austria; e The Institute for Solid State Physics, The University of Tokyo, Kashiwa, Japan; f Department of Life Science and Applied Chemistry, Nagoya Institute of Technology, Showa, Nagoya, Japan; g OptoBioTechnology Research Center, Nagoya Institute of Technology, Showa, Nagoya, Japan; h Limnological Station, Department of Plant and Microbial Biology, University of Zurich, Kilchberg, Switzerland; University of British Columbia

**Keywords:** heliorhodopsin, rhodopsins, metagenomics, oxidative stress

## Abstract

Rhodopsins are light-activated proteins displaying an enormous versatility of function as cation/anion pumps or sensing environmental stimuli and are widely distributed across all domains of life. Even with wide sequence divergence and uncertain evolutionary linkages between microbial (type 1) and animal (type 2) rhodopsins, the membrane orientation of the core structural scaffold of both was presumed universal. This was recently amended through the discovery of heliorhodopsins (HeRs; type 3), that, in contrast to known rhodopsins, display an inverted membrane topology and yet retain similarities in sequence, structure, and the light-activated response. While no ion-pumping activity has been demonstrated for HeRs and multiple crystal structures are available, fundamental questions regarding their cellular and ecological function or even their taxonomic distribution remain unresolved. Here, we investigated HeR function and distribution using genomic/metagenomic data with protein domain fusions, contextual genomic information, and gene coexpression analysis with strand-specific metatranscriptomics. We bring to resolution the debated monoderm/diderm occurrence patterns and show that HeRs are restricted to monoderms. Moreover, we provide compelling evidence that HeRs are a novel type of sensory rhodopsins linked to histidine kinases and other two-component system genes across phyla. In addition, we also describe two novel putative signal-transducing domains fused to some HeRs. We posit that HeRs likely function as generalized light-dependent switches involved in the mitigation of light-induced oxidative stress and metabolic circuitry regulation. Their role as sensory rhodopsins is corroborated by their photocycle dynamics and their presence/function in monoderms is likely connected to the higher sensitivity of these organisms to light-induced damage.

**IMPORTANCE** Heliorhodopsins are enigmatic, novel rhodopsins with a membrane orientation that is opposite to all known rhodopsins. However, their cellular and ecological functions are unknown, and even their taxonomic distribution remains a subject of debate. We provide evidence that HeRs are a novel type of sensory rhodopsins linked to histidine kinases and other two-component system genes across phyla boundaries. In support of this, we also identify two novel putative signal transducing domains in HeRs that are fused with them. We also observe linkages of HeRs to genes involved in mitigation of light-induced oxidative stress and increased carbon and nitrogen metabolism. Finally, we synthesize these findings into a framework that connects HeRs with the cellular response to light in monoderms, activating light-induced oxidative stress defenses along with carbon/nitrogen metabolic circuitries. These findings are consistent with the evolutionary, taxonomic, structural, and genomic data available so far.

## INTRODUCTION

The ability to harness the Sun’s electromagnetic radiation by channeling it into high-energy phosphate bonds empowered microorganisms to tap into an inexpensive and inexhaustible source of energy. Life’s billion-year history of metabolic innovations led to the emergence of only two biological complexes capable of harvesting light: one based on rhodopsins and the other on (bacterio)chlorophyll. Rhodopsins encompass the most diverse and abundant photoactive proteins on Earth and were until recently canonically split between type 1 (microbial rhodopsins) and type 2 (animal rhodopsins) families. Type 1 and 2 rhodopsins families share a similar topological conformation and little or no sequence similarity among each other. Despite dissimilarities in function, structure, and phylogeny, type 1 and 2 rhodopsins have a similar membrane orientation, with their N terminus being situated in the extracellular space. Recently identified during a functional metagenomics screen and characterized by low sequence similarity compared to type 1 rhodopsins, heliorhodopsins (HeRs) have attracted increasing research interest due to their peculiar membrane orientation (i.e., the N terminus in the cytoplasm and the C terminus in the extracellular space) (1), unusual protein structure (2), and controversial taxonomic distribution (3). While electrophysiological (1), physicochemical (4), and structural (2, 5) studies have achieved great progress in elucidating a series of characteristics ranging from photocycle length and lack of ion-pumping activity to detailed protein organization, they provide little information regarding the biological function of HeRs. Moreover, polarized opinions regarding the putative ecological role and taxonomic distribution of HeR-encoding organisms (2, 3) call for the use of novel approaches in establishing HeR functionality. The present study draws its essence from the tenet that functionally linked genes within prokaryotes are coregulated and thus occur close to each other (6, 7). Within this framework, the functions of uncharacterized genes (i.e., HeRs) can be inferred from their genomic surroundings. Here, we couple HeR distributional patterns with contextual genomic information involving protein domain fusions and operon organization and gene expression data to shed light on HeR functionality.

## RESULTS

In order to shed light on the distribution and functional role of HeRs in nature, we conducted a comprehensive survey of genomes and metagenomes enabling us to phylogenetically constrain HeR distribution patterns. Once these patterns were constrained, we evaluated genomic and metagenomic sequences for domain fusions and gene context information in order to identify potential effectors of HeR signaling, identifying potential effector domains and several operons with the potential to couple light sensing to metabolic responses. Finally, to better evaluate the potential for cotranscriptional responses identified *in silico*, we conducted strand-specific metatranscriptomics in a freshwater ecosystem to identify expressed HeRs linked to functional genes.

### Taxonomic distribution.

Previous assessments of taxonomic distribution of HeRs reported conflicting data regarding their presence in monoderm (3) and diderm (2) prokaryotes. In order to accurately map HeR taxonomic distribution, we used the GTDB database (release 89), since it contains a wide-range of high-quality genomes derived from isolated strains and environmental metagenome-assembled genomes, classified within a robust phylogenomic framework (8). By scanning 24,706 genomes, we identified 469 *bona fide* HeR sequences (topology: C-terminal inside and N-terminal outside, seven transmembrane helices and a SxxxK motif in helix 7; see Table S1 [https://doi.org/10.6084/m9.figshare.13286486]) spanned across 17 phyla (out of 151; see Table S2 [https://doi.org/10.6084/m9.figshare.13286486]). In order to assign HeR-containing genomes to either monoderm or diderm categories, we employed a set of 27 manually curated protein domain markers (see Table S13 [https://doi.org/10.6084/m9.figshare.13286486]) that are expected to be restricted to organisms possessing double-membrane cellular envelopes (i.e., diderms) (9). While most analyses were expected to be influenced by various levels of genome completeness, we found that a conservative criterion of presence of at least 10 marker domains singled out all diderm lineages (i.e., *Negativicutes*, *Halanaerobiales*, and *Limnochondria*) (9, 10) within the larger monoderm phylum Firmicutes, apart from correctly identifying other well-known diderms. Except for three genomes (one each belonging to *Myxococcota*, *Spirochaetota*, and *Dictyoglomota* phyla), all other HeR occurrences were restricted to monoderms (see Table S2 [https://doi.org/10.6084/m9.figshare.13286486]). Examination of the HeR-encoding *Myxococcota* contig by querying its predicted proteins against the RefSeq and GTDB databases revealed it to be an actinobacterial contaminant. The *Spirochaeta* genome was incomplete (60% estimated completeness) and only encoded two outer membrane marker genes, making any inferences regarding its affiliation to monoderm or diderm bacteria impossible. However, we could not rule out that this genome could belong to a member lacking lipopolysaccharides (LPS) (9). The *Dictyoglomota* genome belongs to an isolate, and despite its high completeness, it encodes only five markers. Combined with the notion that *Dictyoglomota* are known to have atypical membrane architectures (11), the presence of only five marker points toward the absence of a classical diderm cell envelope. Apart from these exceptions, all other HeR-encoding genomes are monoderm and, at least within this collection, we found no strong evidence of HeRs being present in any organism that is conclusively diderm. We also identified HeRs in several assembled metagenomes and metatranscriptomes (see Materials and Methods). For improved resolution of taxonomic origin, we considered only contigs of at least 5 kb in length (*n* = 1,340 from metagenomes and *n* = 4 from metatranscriptomes). Following a strict approach to taxonomy assignment (i.e., at least 60% genes giving best-hits to the same phylum and not just majority-rule), we could designate a phylum for most HeRs. Without any exception, we found that all the contigs that received robust taxonomic classification (*n* = 1,319) belonged to known monoderm phyla (see Table S3 [https://doi.org/10.6084/m9.figshare.13286486]).

### Domain fusions.

**FIG 1:**
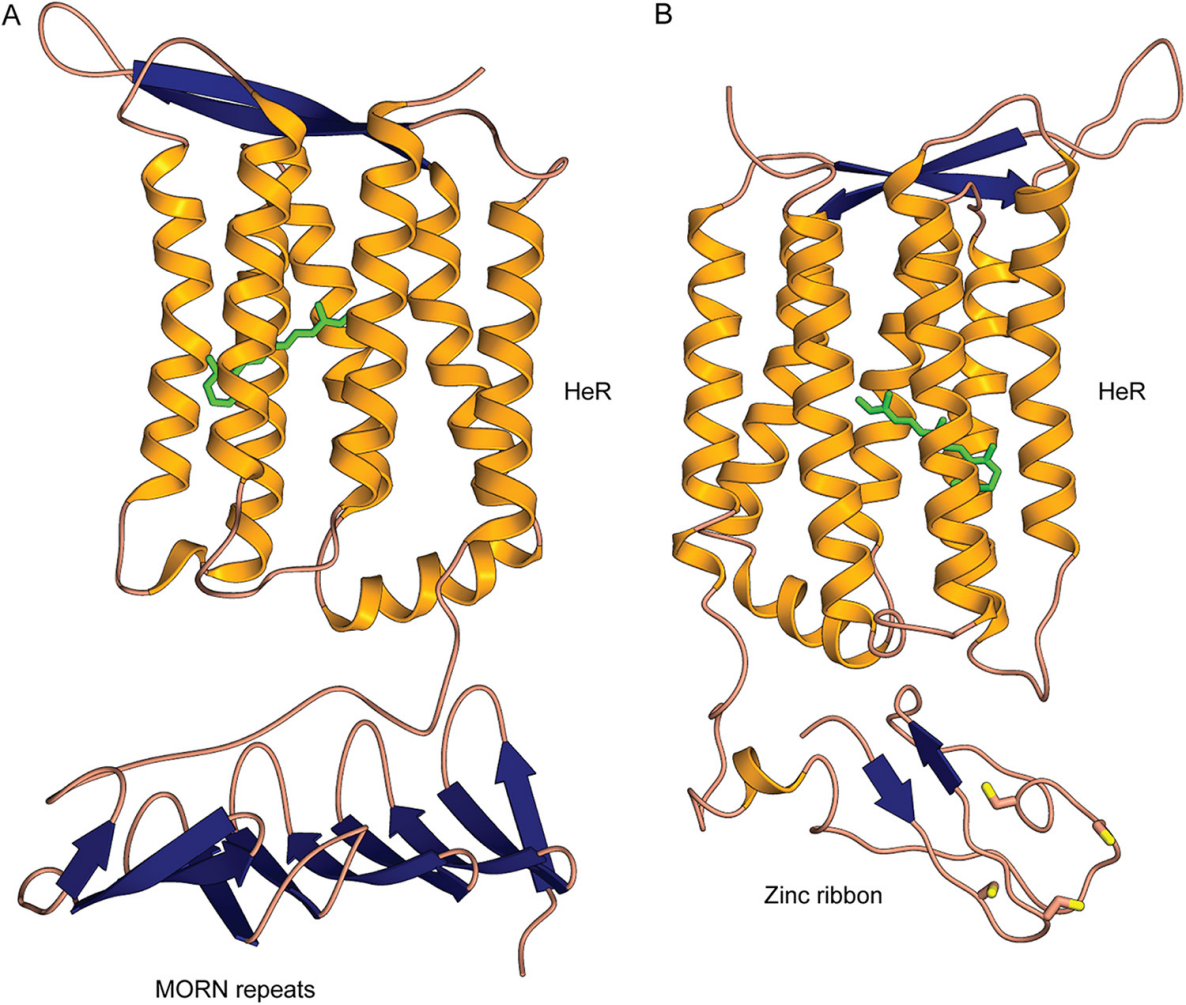
Modeled 3D structures of MORN-HeR and Znf-HeR protein domain fusions. (A) 3D model of a heliorhdodopsin (HeR) containing three N-terminal MORN domain repeats. (B) 3D model of a HeR containing an N-terminal Zn ribbon motif. Both models are oriented with the extracellular side up and the intracellular side down. Retinal is colored green, and cysteine residues are depicted with yellow-topped orange sticks.

Domain fusions with rhodopsins are recently providing novel insights into the diverse functional couplings that enhance the utility of a light sensor, e.g., the case of a phosphodiesterase domain fused with a type 1 rhodopsin (12). As far as we are aware, no domain fusions have yet been described for HeRs. In our search for such domain fusions that may shed light on HeR functionality, the MORN repeat (Membrane Occupation and Recognition Nexus; PF02493) was found in multiple copies (typically 3) at the cytoplasmic N terminus of some HeRs (*n* = 36). A tentative three-dimensional (3D) model for a representative MORN-HeR could be generated and is shown in Fig. 1A.

**FIG 2:**
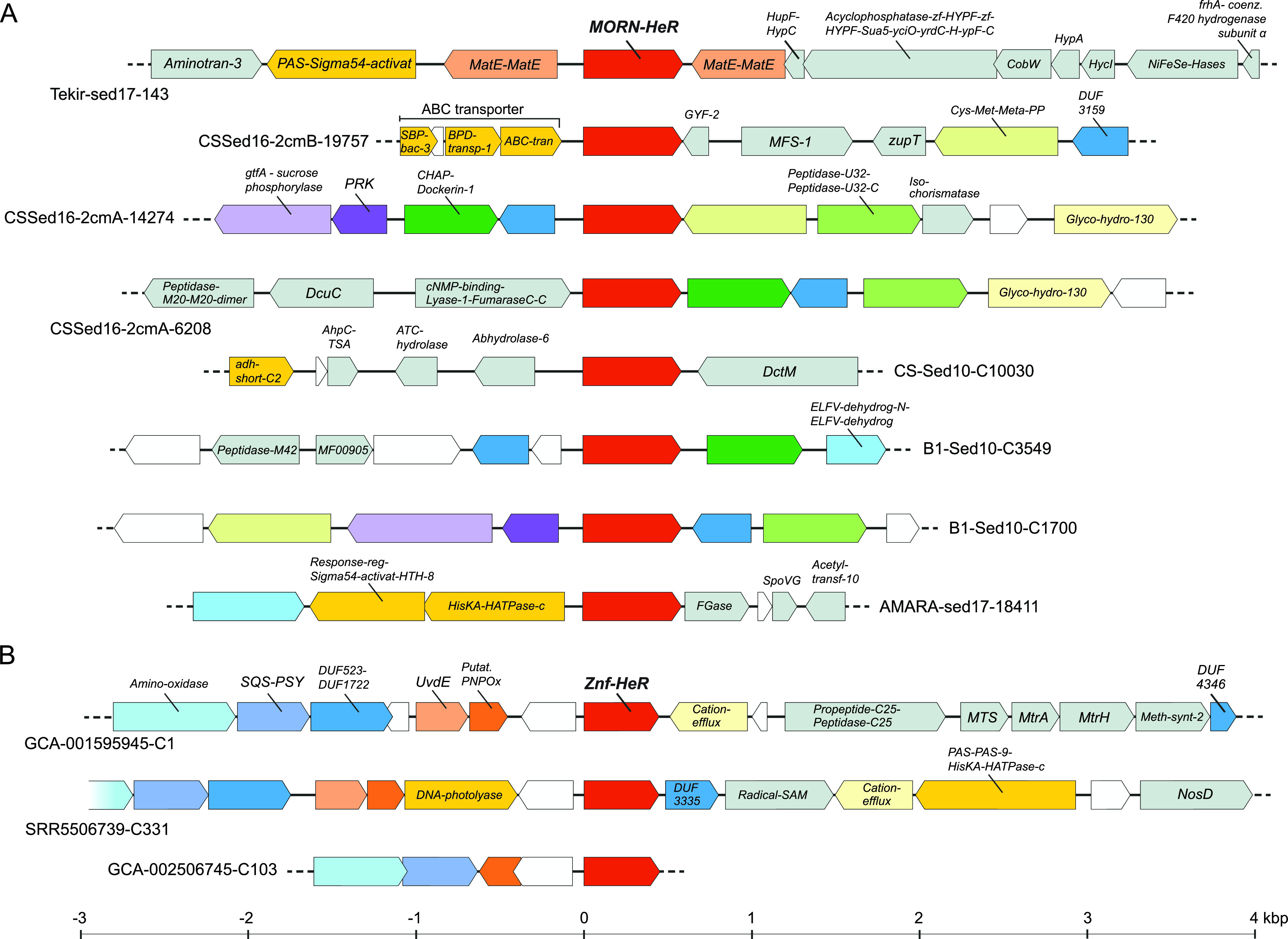
Genomic context of HeR-protein domain fusion genes. (A) Representative MORN-HeR encoding contigs identified in strictly anaerobic *Firmicutes.* (B) Contigs encoding Znf-HeR domain fusions. Neighboring genes were depicted within an interval spanning ∼7 kb, centered on HeR. Genes occurring only once within the considered intervals are colored gray; genes encoding HisKA, PAS, and regulatory domains, as well as other discussed HeR neighbors, are depicted in bright yellow. Homologous genes occurring multiple times found within each category of HeR-protein fusion contigs are depicted using matching colors. Hypothetical genes are white.

These MORN-HeR sequences were phylogenetically restricted to two environmental branches of metagenome-assembled genomes (MAGs) recovered from haloalkaline sediments that affiliate to the family *Syntrophomonadaceae* (phylum *Firmicutes*) (13–15) (see Fig. S1 [https://doi.org/10.6084/m9.figshare.13286486]). The prototypic MORN repeat, consisting of 14 amino acids with the consensus sequence YEGEWxNGKxHGYG, was first described in 2000 (16) from junctophilins present in skeletal muscle and later recognized to be ubiquitous in both eukaryotes and prokaryotes (17). This conserved signature can be seen in the alignment of MORN-repeats fused to HeRs (see Fig. S2 [https://doi.org/10.6084/m9.figshare.13286486]). MORN-repeats have been shown to bind to phospholipids (18, 19), promoting stable interactions with plasma membranes (16) and also function as protein-protein interaction modules involved in di- and oligomerization (20). They are expected to be intracellular and provide a large putative interaction surface (either with other MORN-HeRs or other proteins). A widespread adaptation of bacteria to alkaline environmental conditions is the increased fluidity of their plasma membranes achieved by the incorporation of branched-chain and unsaturated fatty acids, which ultimately influences the configuration and activity of membrane integral proteins such as ATP synthases and various transporters (21). Microbial rhodopsins typically associate as oligomers *in vivo*, which is also the case with heliorhodopsins that are known to form dimers (5, 22). Indeed, it has been demonstrated that lipid composition of the membrane can directly affect proteorhodopsin dimerization (23). The presence of MORN-repeats in HeRs exclusively within extreme haloalkaliphilic bacteria (class *Dethiobacteria*) may be accounted for via their potential role in stabilizing HeR dimers in conditions of increased membrane fluidity (see Fig. S4 [https://doi.org/10.6084/m9.figshare.13286486]). Another possibility would be the interaction of MORN-repeats with other MORN-repeat containing proteins encoded in these MAGs. We could indeed identify multiple MORN-protein domain fusions co-occurring in genomes of analyzed *Dethiobacteria* (see Fig. S1 and S3 and Table S15 [https://doi.org/10.6084/m9.figshare.13286486]). Even though the nature of interactions among these proteins with intracellular MORN-repeats is unclear, they raise the possibility that MORN-repeats act as downstream transducers of conformational changes occurring in HeRs. Such tandem repeat structures may function as versatile target recognition sites capable of binding not only small molecules like nucleotides but also peptides and larger proteins (24). If true, this would render HeRs as sensory rhodopsins. In support of this, we found several genes in close proximity to MORN-HeRs encoding signature protein domains (e.g., PAS, HisKA, and HATPase_c) that are known to be involved in histidine kinase signaling (25) (Fig. 2A).

Since no other obvious domains were found to be fused with HeRs using standard profile searches, we examined all N- and C-terminal extensions, as well as loops longer than 50 amino acids, by performing more sensitive profile-profile searches using HHpred (26). We found at least 10 N-terminal extensions of HeRs (ntv1 to ntv10), 22 variants of ECL1 (extracellular loop 1), a single type of loop extension for ICL2 (intracellular loop 2), and three variants of ICL3 (intracellular loop 3). A complete listing of all alignments and summary results of HHpred can be found in Table S8 (https://doi.org/10.6084/m9.figshare.13286486). Remarkably, we found significant matches in a set of six sequences (all originating from *Thermoplasmatales* archaea) to zinc ribbon proteins (Pfam domain zinc_ribbon_4) at the N terminus of some heliorhodopsins (these extensions are termed N-terminal variant 1 or ntv1; see Table S8 [https://doi.org/10.6084/m9.figshare.13286486]). Zinc ribbons belong to the larger family of zinc-finger domains (27). A CxxC-17x-CxxC was found in this region that likely coordinates a metal (e.g., zinc or iron). These CxxC_CxC-type motifs are common to a wider family of zinc-finger-like proteins that were initially found to bind to DNA and later shown to be capable of binding to RNAs, proteins, and small molecules (27). Similar motifs are also seen in rubredoxins and Cys_rich_KTR domains. We term these fused ntv1 protein variants as Znf-HeRs (zinc-finger heliorhodopsins). A modeled structure for a representative Znf-HeR is shown in Fig. 1. In one contig encoding a Znf-HeR we identified a histidine kinase that could be functionally linked (Fig. 2B). Notably, most identified Znf-HeRs are flanked by genes known to be triggered by light exposure and play key roles in photoprotection (i.e., the carotenoid biosynthesis genes, e.g., lycopene cyclase, phytoene desaturase, amino oxidase, and squalene/phytoene synthase [SQS-PSY]) and UV-induced DNA damage repair (DNA photolyases and UV-DNA damage endonucleases [UvdE]) (28, 29). Recent research showed that HeRs from *Thermoplasmatales* archaea (*Ta*HeR) and uncultured freshwater *Actinobacteria* (48C12) (for which the structure is resolved and lacks the ntv1 extension) might bind zinc (30). Since the zinc binding site could not be precisely identified, it was suggested that it could be located in the cytoplasmic part and responsible for modifying the function of HeR. Our discovery of Znf-HeRs offers additional, more direct indications of the role of zinc in the possible downstream signaling by HeRs.

### Gene context analysis.

**FIG 3:**
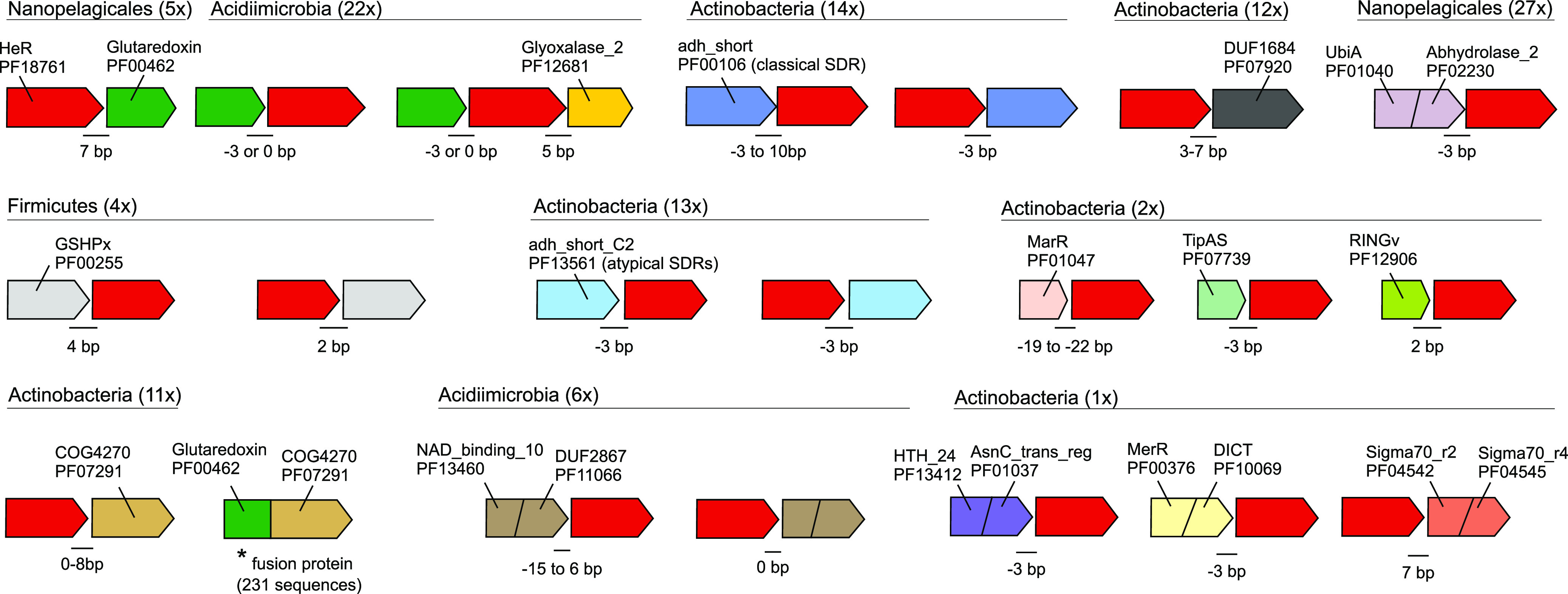
Schematic representation of genes that may be transcriptionally linked to HeRs. Taxonomic categories and number of occurrences are shown at the top of each putative operon. Intergenic distances (in bp) are indicated at gene junctions. Negative distance values indicate overlapping genes. Pfam or COG identifiers are used to represent domain architectures. An asterisk (*) indicates a fused gene (two domains: glutaredoxin and COG4270) found in at least 473 genomes from GTDB and 231 unique sequences in UniProt, suggesting a functional linkage of COG4270 with glutaredoxin.

We reasoned that aside from domain fusions that represent a more direct functional association, gene context analyses, i.e., the repeated presence of specific genes/domains in close proximity to HeRs, may also provide additional clues toward linkage with specific functions. Such linkage may take the form of potential operons or overrepresented genes in the HeR neighborhood. Given the large number of long contigs encoding HeRs (from genomes and metagenomes), we sought to identify candidate genes that could be transcribed together with HeRs (in the same operon). We used the following strict criteria for obtaining such genes: (i) the intergenic distance between such a gene and the HeR must be <10 bp, and (ii) the gene must be located on the same strand. A number of interesting candidates emerged in this analysis with the most frequent ones being summarized in Fig. 3 (for a complete table, see Table S9 [https://doi.org/10.6084/m9.figshare.13286486]).

We identified multiple instances in which genes with glutaredoxin and GSHPx PFAM domains were found adjacent to HeRs (*n* = 31). Glutaredoxins are small redox proteins with active disulfide bonds that utilize reduced glutathione as an electron donor to catalyze thiol-disulfide exchange reactions. They are involved in a wide variety of critical cellular processes such as the maintenance of cellular redox state, iron and redox sensing, and the biosynthesis of iron-sulfur clusters (31, 32). Glutathione is also used by glutathione peroxidase (GSHPx) to reduce hydrogen peroxide and peroxide radicals, i.e., as an antioxidative stress protection system (33). In addition, there are also instances where glutaredoxin and genes containing glyoxalase_2 domains may be cotranscribed with HeRs. Glyoxalases, in concert with glutaredoxins, are critical for the detoxification of methylglyoxal, a toxic by-product of glycolysis (34). Moreover, adjacent to HeRs we find at least three instances where a catalase gene is also present (in *Actinobacteria*; see Fig. S10 and S11 [https://doi.org/10.6084/m9.figshare.13286486]). Collectively, these observations suggest a role for HeRs in oxidative stress mitigation. In one case, we found a gene encoding the DICT domain (Fig. 3), which is frequently associated with GGDEF, EAL, HD-GYP, STAS, and two-component system histidine kinases. Notably, it has been predicted to have a role in light response (25).

### Strand-specific metatranscriptomics.

Although we assembled contigs encoding HeRs from previously published metatranscriptomes, the lack of strand-specific transcriptomes hampered any clear conclusions on whether or not genes adjacent to HeRs are indeed cotranscribed, leaving open the possibility that they might simply be artifacts of assembly (35). In order to gather more definitive evidence for cotranscription of HeRs with neighboring genes, we performed strand-specific metatranscriptome sequencing for a freshwater sample (see Materials and Methods). The freshwater habitat was chosen because HeRs are widely distributed in these habitats and in particular in freshwater *Actinobacteria* (from which they were originally described) (1). In addition, *Actinobacteria* being among the most abundant microbes in these habitats (36, 37) would increase the chances for recovery of such polycistronic transcripts.

**FIG 4:**
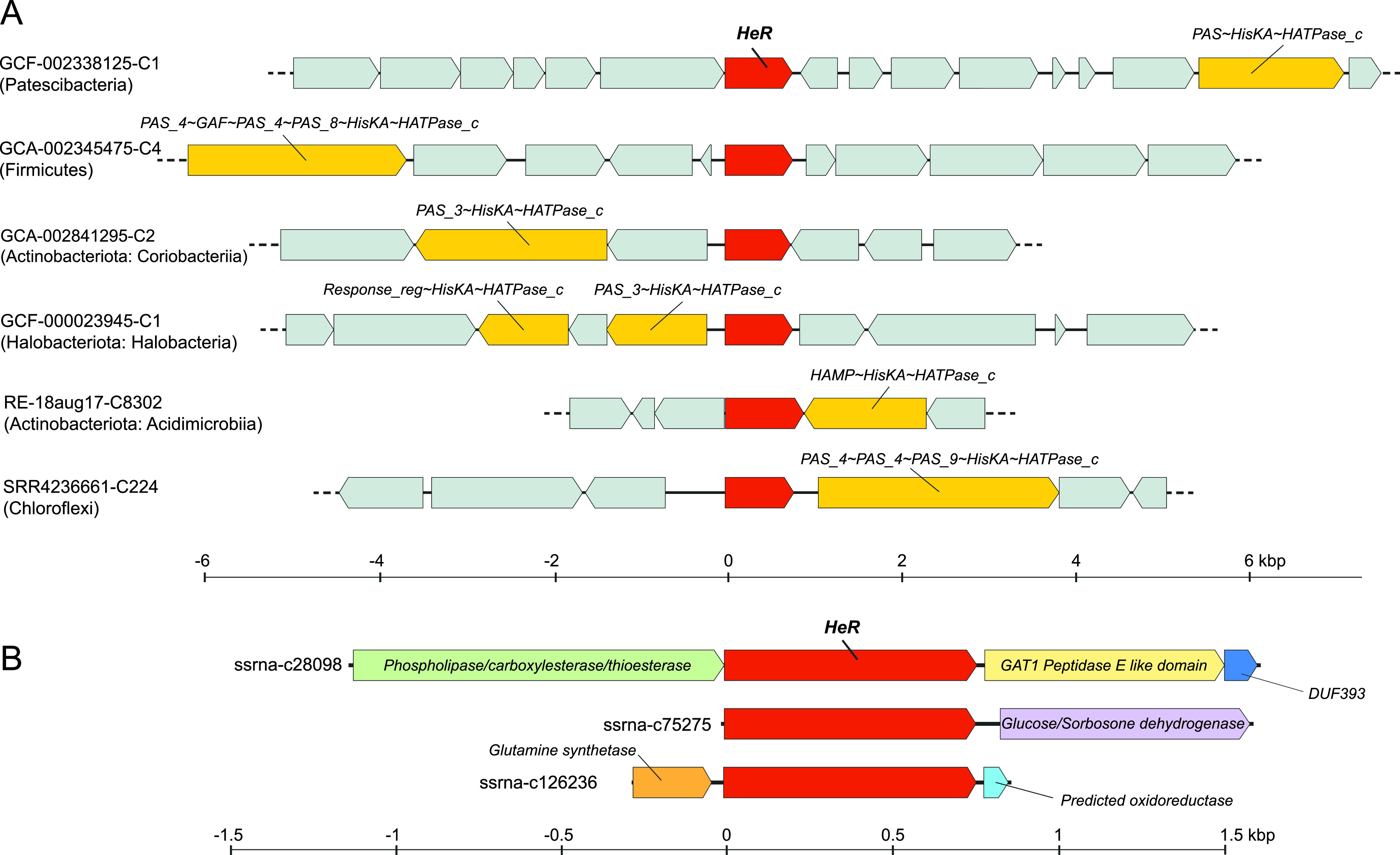
Selected HeR gene contexts. (A) Genes encoding HisKA domain signaling proteins identified in the proximity of HeR genes from diverse phyla. All genes containing HisKA domains are colored bright yellow, HeRs are shown in red, and all other genes are indicated in gray. (B) Transcripts obtained by strand-specific metatranscriptomics from freshwater encoding genes coexpressed with HeR.

We recovered six HeR-encoding transcripts that were >1 kb in length. All these transcripts are predicted to originate from highly abundant freshwater *Actinobacteria* with streamlined genomes (four transcripts from “*Ca.* Planktophila” and two from “*Ca.* Nanopelagicus”) (see Table S12 [https://doi.org/10.6084/m9.figshare.13286486]) (37). Overall, there are three types of transcripts based upon gene content: (i) class1, encoding glutamine synthetase catalytic subunit and NAD^+^ synthetase; (ii) class 2, encoding a hydrolase, a peptidase, and a DUF393 domain containing protein; and (iii) class 3, encoding glucose/sorbosone dehydrogenase (GSDH) (Fig. 4B; see also Table S12 [https://doi.org/10.6084/m9.figshare.13286486]). A common theme for glutamine synthetase and NAD^+^ synthetase is that both utilize ammonia and ATP to produce glutamine and NAD^+^, respectively. Moreover, some NAD^+^ synthetases may be glutamine dependent (38). Glutamine synthetase in particular is a key enzyme for nitrogen metabolism in prokaryotes at large (39). For hydrolases and peptidases, the function prediction is somewhat broad. Glucose/sorbosone dehydrogenase catalyzes the production of gluconolactone from glucose (40). Therefore, it appears that all six HeRs are generally cotranscribed with genes involved in nitrogen assimilation and degradation/assimilation of sugars and peptides. This would suggest that these processes are also influenced by light, with such a link between light-dependent increase in sugar uptake and metabolic activity being recently proposed in nonphototrophic *Actinobacteria* (41). Light also triggers photosynthetic activity, increasing the availability of sugars and other nutrients (e.g., glutamine and ammonia) for heterotrophs. In this vein, a link between a light sensing mechanism, e.g., via heliorhodopsins, may lead to elevated metabolic activity.

In a previous study, histidine kinases were deemed absent in the vicinity of HeRs (2). Given that our initial analyses predicted a sensory function, we examined genomic regions spanning 10 kb up- and downstream of HeRs. Already in the case of MORN-HeRs and Znf-HeRs, we observed histidine kinase signaling components in close proximity to them (Fig. 2). In our search we detected multiple instances of histidine kinases (HisKA) fused with PAS, GAF, MCP_Signal, HAMP, or HATPase_c domains in the gene neighborhoods of HeRs in distinct phyla (e.g., *Actinobacteria*, *Chloroflexi*, *Patescibacteria*, *Firmicutes*, *Dictyoglomota*, and *Thermoplasmatota*) (Fig. 4B; for more details, see Fig. S4 to S15 [https://doi.org/10.6084/m9.figshare.13286486]). Moreover, in many cases multiple response regulator genes were present in the same regions (Pfam domains Response_reg and Trans_reg_C). Less frequently, GGDEF and EAL domains, usually associated with bacterial signaling proteins, were also present. Using overrepresentation analysis (42), we found that the occurrence of two-component system protein domains in the vicinity of HeRs is statistically significant (see Materials and Methods and Table S11 [https://doi.org/10.6084/m9.figshare.13286486]). In addition to these two-component system proteins, the same regions also appear enriched in redox proteins (e.g., thioredoxin, peroxidase, and catalase). The close association of two-component systems, genes involved in oxidative stress mitigation and HeRs points toward a functional interaction.

## DISCUSSION

Contextual genomic information shows that monoderm prokaryotes use HeRs in multiple mechanisms for the activation of downstream metabolic pathways after light sensing. These observations offer tantalizing clues regarding the involvement of HeRs in multiple cellular processes and add new lines of inquiry for the primary role of HeRs in light-activated signal transduction. Additional support for the role of HeRs in light sensing is inferred from the frequent association of HeRs with classical histidine kinases and associated protein domains in multiple phyla. Furthermore, multiple types of N-terminal domain fusions found in specific subfamilies of HeRs (i.e., MORN domains in haloalkaliphilic *Firmicutes* and zinc-ribbon-type domains in *Thermoplasmatales* archaea) point to possible downstream signaling which may be effected by the recruitment of additional, as-yet-unknown, partner proteins.

We further propose a critical role for HeRs in protecting monoderm cells from light-induced oxidative damage. In this sense, we observed a close association and probable transcriptional linkage of HeRs to glyoxylases and glutaredoxins (sometimes seen as overlapping genes). Given that light can induce the uptake and metabolism of sugars, as previously discussed for certain *Actinobacteria* (41), it is expected that increased sugar availability resulting from photosynthesis leads to increased glycolytic activity in heterotrophic bacteria. Glycolysis also produces small amounts of toxic methylglyoxal that can be neutralized by the combined action of glyoxylases and glutaredoxins. In this sense, it appears that at least in some *Actinobacteria* glyoxylases and glutaredoxins may be transcribed together with HeRs, but how the transcription is controlled remains unclear. Additional evidence of transcriptional linkages of HeRs to proteins like peroxiredoxin and catalase also imply a light-dependent activation, boosting the cellular response to light induced oxidative damage which may be critical for both aerobes and anaerobes. Evidence from strand-specific HeR transcripts originating from freshwater *Actinobacteria* suggests the further involvement of HeRs in nitrogen and sugar metabolism via glutamate synthase, NAD^+^ synthases, and glucose/sorbosone dehydrogenases in these organisms. However, direct experimental evidence of interactions of HeRs with the genes proposed here could take multiple forms, e.g., strand-specific transcriptomics data from cultured microbes that encode HeRs supplemented with similar data from diverse environments, and the use of HeR knockouts combined with transcriptomic data under conditions of light and dark.

Overall, the picture that emerges (at least for some organisms) is one of HeR’s roles in responding to light and transmitting the signal via histidine kinases. Downstream processes that are ultimately regulated are diverse, including possible roles for HeRs in the mitigation of light-induced oxidative damage and in the regulation of nitrogen assimilation and carbohydrate metabolism, processes that may benefit from a light-dependent activation through more efficient utilization of available resources.

Recent work has shown more support for the diderm-first ancestor (43) and, given the far broader distribution of type 1 rhodopsins in both mono- and diderm organisms, it appears likely that type 1 rhodopsins emerged prior to HeRs. The very restricted distribution of HeRs to monoderms would support this view as well. Even so, HeRs are not universally present in monoderms and, when present, appear to be associated with diverse genes involved in signal transduction, oxidative stress mitigation, and nitrogen and glucose metabolism. This suggests they have been exapted as generalized sensory switches that may allow light-dependent control of metabolic activity in multiple lineages, somewhat similar to type 1 rhodopsins where minor modifications have led to emergence of a wide variety of ion pumps (44). The frequent distribution of HeRs in aquatic environments (habitats characterized by increased light penetration), where they commonly occur within phylum *Actinobacteriota*, helps us to explain their monoderm-restricted presence. Abundant freshwater actinobacterial lineages are generally typified by lower GC content (45) and increased vulnerability to oxidative stress damage (46). This susceptibility is also illustrated by actinobacterial phages that exhibit positive selection toward reactive oxygen species defense mechanisms (36). This suggests that oxidative stress is a considerable influence in environment at large, and it has indeed been identified as such before (47, 48). Light-induced, oxygen-dependent inactivation has also been demonstrated in other bacterial species as well (49). Such inactivation is understood to be the direct result of the production of reactive oxygen species by endogenous porphyrins in the presence of light (50, 51). Moreover, reactive oxygen species are also released by other community members and generated by UV-induced photochemical reactions (47). Given the fact that monoderms are generally more sensitive to light-induced damage (52) and taken together with the above-mentioned metabolic implications, we consider that HeRs evolved as sensory switches capable of triggering a fast response against photo-oxidative stress in prokaryotic lineages more sensitive to light.

## MATERIALS AND METHODS

### Metagenomes and metatranscriptomes.

We used previously published metagenomics and metatranscriptomics data from freshwaters (36, 53, 54), haloalkaline brine and sediment (14, 15), brackish sediments (55), a GEOTRACES cruise (56) and TARA expeditions (57). In addition, we downloaded multiple environmental metagenomes (sludge, marine, pond, estuary, etc.) from EBI MGnify (https://www.ebi.ac.uk/metagenomics/) (58) and assembled them using Megahit v1.2.9 (59). All contigs in this work are named or retain existing names that allow tracing them to their original data sets.

### Sequence search for *bona fide* rhodopsins.

Genes were predicted in metagenomics contigs using Prodigal (60). Candidate rhodopsin sequences were scanned with hmmsearch (61) using PFAM models (PF18761, heliorhodopsin; PF01036, bac_rhodopsin), and only hits with significant E values (<1E–3) were retained. Homologs for these sequences were identified by comparison to a known set of rhodopsin sequences (55) using MMSeqs2 (62), and alignments were made using MAFFT-linsi (63). These alignments were used as input to Polyphobius (64) for transmembrane helix prediction. Only those sequences that had seven transmembrane helices and either a SxxxK motif (for heliorhodopsins) or DxxxK motif (for proteorhodopsins) in TM7 were retained. In addition, we also screened the entire UniProtKB for HeRs. In total, we accumulated at least 4,108 (3,606 + 502) *bona fide* HeR sequences.

### Taxonomic classification of assembled contigs.

Contigs were dereplicated using cd-hit (65) (95% sequence identity and 95% coverage). Only contigs ≥5 kb were retained for this analysis. A custom protein database was created by predicting and translating genes in all GTDB genomes (release 89) (8) using Prodigal (60). These sequences were supplemented with viral and eukaryotic proteins from UniProtKB (66). Best hits against predicted proteins in contigs were obtained using MMSeqs2 (62). Taxonomy was assigned to a contig (minimum length, 5 kb) only if ≥60% of genes in the contig gave best hits to the same phylum. All contigs that appeared to originate from diderms were cross-checked against NCBI RefSeq (accessed online on 15 December 2020).

### Outer-envelope detection.

A set of protein domains found in genes encoding the outer-envelope (9) was further reduced to include only those domains that were found mostly in known diderms. These domains were searched against the predicted proteins in all genomes in GTDB using hmmsearch (E value < 1E–3). The results are shown in Table S13 (https://doi.org/10.6084/m9.figshare.13286486).

### Protein function-structure predictions.

Predicted proteins were annotated using TIGRFAMs (67) and COGs (68). Domain predictions were carried out using the pfam_scan.pl script against the PFAM database (release 32) (17). Profile-profile searches were carried out online using the HHPred server (26). Additional annotations were added using Interproscan (69). Protein structure predictions were carried out using the Phyre2 server (70), and structures were visualized with CueMol (http://www.cuemol.org/en/).

### Domains overrepresentation near heliorhodopsin.

A subset of high-quality MAGs (*n* = 240) containing HeR-encoding genes flanked both up- and downstream by a minimum of 10 genes were selected from GTDB (release 89) (8). For each genome, the probability of finding any particular domain by chance in a random subset of 20 genes was calculated using the hypergeometric distribution (without replacement) in R with the function *phyper* (Stats package) (71). In order to account for type I errors arising from multiple comparisons, hypergeometric test *P* values were adjusted using the Benjamini-Hochberg procedure (72). Further, we selected domains located in the proximity of HeR in at least 10% of genomes with low probability (false discovery rate corrected *P* value < 0.05). This procedure that was initially employed for the whole GTDB genome collection was repeated for individual phyla containing HeR-encoding genes within at least five genomes.

### Strand-specific freshwater transcriptome sequencing and assembly.

Sampling was performed on the 16th of August 2020 at 9:00 in Řimov Reservoir, Czech Republic, (48°50′54.4″N, 14°29′16.7″E) using a hand-held vertical Friedinger (2 L) sampler. A total of 20 L of water were collected from a depth of 0.5 m and immediately transported to the laboratory. Serial filtration was carried out by passing water sample through a 20-μm-pore-size prefilter mesh, followed by a 5-μm-pore-size PES filter (Sterlitech) and a 0.22-μm-pore-size PES (polyethersulfone) filter (Sterlitech, USA) using a Masterflex peristaltic pump (Cole-Palmer, USA). Filtration was done at maximum speed for 15 min to limit cell lysis and RNA damage as much as possible. A total volume of 3.7 L was filtered during this time. PES filters (5-μm and 0.22-μm pore sizes) were loaded into cryo-vials prefilled with 500 μl of DNA/RNA Shield (Zymo Research, USA) and stored at −80°C. RNA was extracted from filters using the Direct-zol RNA MicroPrep (Zymo Research) after they had been previously thawed, partitioned, and subjected to mechanical lysis by bead beating in ZR BashingBead lysis tubes (with 0.1- and 0.5-mm spheres). DNase treatment was performed to remove genomic DNA during RNA extraction as an “in-column” step described in the Direct-zol protocol and was repeated after RNA elution, by using the Ambion Turbo DNA-free kit (Life Technologies, USA). RNA was quantified using a NanoDrop ND-1000 UV-Vis spectrophotometer (Thermo Fisher Scientific, USA), and integrity was verified by agarose gel (1%) electrophoresis. A total of 4.6 μg of RNA from the 0.22-μm-pore-size filter and 2.6 μg from the 5-μm-pore-size filter were sent for dUTP-marking based strand-specific metatranscriptomic sequencing at Novogene. Following quality control at Novogene, the samples were mixed into a single reaction, subjected to rRNA depletion, and used for stranded library preparation. Strand specificity was achieved by the incorporation of dUTPs instead of dTTPs in the second-strand cDNA, followed by digestion of dUTPs by uracil-DNA glycosylase to prevent PCR amplification of this strand. Paired-end (PE 150 bp) sequencing was carried out using a Novaseq 6000 platform. A total of 166,213,184 raw sequencing reads, amounting to 24.9 Gb, were produced. *De novo* assembly of metatranscriptomic data was performed using rnaSPAdes v3.14.1 (73) in reverse-forward strand-specific mode (–ss rf) with the custom k-mers list 29, 39, 49, 59, 69, 79, 89, 99, 109, 119, and 127. A total of 156,235 hard-filtered transcripts of a minimum length of 1 kb were assembled. Protein coding sequences were predicted *de novo* using Prodigal (60) in metagenomic mode (-p meta). Protein domains were annotated by scanning with InterProScan (69), while PFAM (Protein Families) (17) domains were identified using the publicly available Perl script pfam_scan.pl (ftp://ftp.ebi.ac.uk/pub/databases/Pfam/Tools/). Proteins were scanned locally using HMMER3 (61) against the COGs (Clusters of Orthologous Groups) (68) HMM database (E value < 1E–5) and the TIGRFAMs (TIGR Families) (67) HMM collection with trusted score cutoffs. BlastKOALA (74) was used to assign KO identifiers (KO numbers). Annotations for representative transcripts encoding HeR are summarized in Table S12 (https://doi.org/10.6084/m9.figshare.13286486).

### Data availability.

Sequence data generated in this study have been deposited in the European Nucleotide Archive (ENA) at EMBL-EBI under project accession number PRJEB35770 (run ERR5100021). The derived data that support the findings of this paper are available in FigShare (https://doi.org/10.6084/m9.figshare.13286486). All other relevant data supporting the findings of this study are available within the paper and its supplementary information files. The R code used for statistical analyses is available in FigShare (https://doi.org/10.6084/m9.figshare.13286486).
